# Textbook outcome in oncological gastric surgery: a systematic review and call for an international consensus

**DOI:** 10.1186/s12957-023-03166-8

**Published:** 2023-09-12

**Authors:** Silvia Carbonell-Morote, Han-Kwang Yang, Javier Lacueva, Juan Jesús Rubio-García, Lucia Alacan-Friedrich, Lea Fierley, Celia Villodre, Jose M. Ramia

**Affiliations:** 1 Department of Surgery, Hospital General Universitario Dr. Balmis, Avenida Pintor Baeza, 11, 03010 Alicante, Spain; 2 ISABIAL, Alicante, Spain; 3 Department of Surgery and Cancer Research Institute, Seoul National University College of Medicine, Seoul, Korea; 4 Universidad Miguel Hernández, Alicante, Spain; 5 Hospital General Universitario de Elche, Elche, Spain; 6 Humboldt University, Berlín, Germany

**Keywords:** Textbook outcome, Postoperative complications, Gastric cancer, Benchmarking

## Abstract

**Background:**

Textbook outcome (TO) is a multidimensional measure used to assess the quality of surgical practice. It is a reflection of an “ideal” surgical result, based on a series of benchmarks or established reference points that may vary depending on the pathology in question. References to TO in the literature are scarce, and the few reports that are available were all published very recently. In the case of gastric surgery, there is no established consensus on the parameters that should be included in TO, a circumstance that prevents comparison between series.

**Aim:**

To present a review of the literature on TO in gastric surgery (TOGS) and to try to establish a consensus on its definition.

**Material and methods:**

Following the PRISMA guide, we performed an unlimited search for articles on TOGS in the MEDLINE (PubMed), EMBASE and Cochrane, Latindex, Scielo, and Koreamed databases, without language restriction, updated on December 31, 2022. The inclusion criterion was any type of study assessing TO in adult patients after oncological gastric surgery. Selected studies were assessed, and TOGS was measured. The parameters used to assess the achievement of TOGS in selected studies were also recorded.

**Results:**

Twelve articles were included, comprising a total of 44,581 patients who had undergone an oncological gastric resection. The median rate of TOGS was 38.6%. All the publications but one included mortality as a TO variable, showing statistically significant differences in favor of the group in which TOGS was achieved. All articles included the number of nodes examined in the surgical specimen, with the assessment of fewer than 15 being associated with a low rate of TOGS achievement in five studies (41.7%). The variable postoperative complications according to the Clavien-Dindo score was the most important cause of failure to achieve TOGS in four studies (33.3%). Seven articles (58.3%) found a significant increase in long-term survival in patients who obtained TO. Advanced age, elevated ASA, and Charlson score had a negative impact on obtaining TOGS.

**Conclusions:**

The standardization of TOGS is necessary to be able to establish comparable results between groups.

## Introduction

Textbook outcome (TO) is an indicator that combines a series of perioperative parameters that together define patients with an ideal postoperative course, and shows the percentage of patients in which this result is achieved [1]. The concept of TO was defined for the first time by Kolfschoten et al. in the field of colorectal cancer surgery and was based on six parameters: hospital stay, mortality, reoperation rate, readmission rate, R0, and surgery without stoma. Since then, several articles have been published on TO in various cancers requiring surgery, using definitions that have sometimes included variables other than the ones just listed [1–5].

The results published are quite heterogeneous because the parameters included to assess TO have varied according to the pathology; sometimes, the parameters used have varied even inside the same pathology, as in the case of gastric carcinoma, for example. Consequently, although TO is a very useful tool for assessing the quality of surgical treatment, the variation in the parameters included makes it hard to obtain valid results and conclusions [6, 7].

Esophagogastric surgery has particularly high morbidity and mortality rates. The first definition of TO in esophagogastric surgery was published by Busweiler in 2017 [1]. Without any doubt, it is one of the most demanding: it includes 10 variables, and the rates of TO obtained are much lower than those recorded in other cancers. Since Busweiler et al.’s study, the definitions of TO in gastric cancer surgery have consistently included variables such as R0 resection and the number of nodes analyzed (15 or more) [2]. However, there are variations in the definition of severe complications, hospital stay, and the period of time (i.e., 30 or 90 days) considered to assess postoperative mortality. In addition, the results of TO for all types of esophagogastric cancer surgery have frequently been presented together, in spite of the fact that they are procedures with different morbidity and mortality rates. Further, in some studies, the main objective is the comparison of high and low-volume hospitals (3,10), but in others, the focus is on the assessment of long-term survival associated with TO (1,12) [3]. Finally, after esophageal and gastric cancer surgery, a direct correlation was observed between the achievement of TO and survival [4].

Thus, TO is regarded as a high-quality indicator for measuring the results of gastric surgery (GS) and as an indicator of long-term survival. We therefore carried out a systematic review of the published literature on TO in gastric cancer surgery (TOGS) with the aim of proposing a set of common variables that make it possible to compare the results of different centers.

## Methods

Adhering to PRISMA guidelines, we performed an unlimited search for articles on TOGS in the MEDLINE (PubMed), EMBASE and Cochrane database, Latindex, Scielo, and Koreamed databases, with no language restriction, updated on December 31, 2022. The search items were ((Textbook outcomes) or (Textbook outcome)) and ((Gastric) or (Stomach)) and ((Surgery)).

The sole inclusion criterion was any type of article that included adult patients in whom TO had been measured after any type of surgery for gastric cancer. Exclusion criteria were studies that combined different types of surgery (gastric and esophageal) without presenting the data for gastric surgery separately, benign gastric surgery, series of pediatric patients, duplicated series, surveys, and editorials.

The following data from the selected studies were included, if available: the author of the study, year of publication, type of study, the Ottawa-Newcastle scale score [5], number of patients included, disease, procedure (type of gastrectomy), percentage of TO, factors associated with achieving TO, the parameter least frequently achieved in obtaining TO, variation between hospitals included in multicenter studies, the relationship between TO and survival, and others (Table 1). The parameters used to define the achievement of TOGS in the selected studies were also recorded (Table 2).

**Table 1:** The median TOGS obtained in population studies

Author/year Journal	Type of study/type of cancer	Number GC Patients	Data: - Treatment/gastrectomy - Neoadjuvant - Approach - Other	TO	Factors associated with TO	Worst contributing factor TO	Variation between hospitals	Survival TO vs no TO/other
Busweiler Br J Surg [1]	National Multicentre Database (Dutch Upper Gastrointestinal Cancer Audit) Esophageal and gastric cancer	1772	**Type of treatment:** total gastrectomy, partial gastrectomy, other gastrectomy, no resection, unknown **Neoadjuvant therapy**: none, chemotherapy, chemoradiotherapy, unknown/other **Surgical approach:** open, laparoscopy, unknown **Timing of surgery:**: elective, emergency **Additional resection owing to tumor invasiveness:** no, yes, unknown	32.1%	ASA grade < 3 Charlson < 2 Clinical tumor stage < 3 Tumor location: corpus, antrum/pylorus Neoadjuvant therapy No additional organ resection	< 15 lymph nodes examined	11.4 to 52.4%	
Priego J Laparoend Adv Surg Tech [8]	Spanish Unicentric Database Gastric cancer	96	**Type of gastrectomy:** total, distal **Neoadjuvant therapy:** yes/no **Type of surgery:** open, laparoscopy	51.04%		Severe complications (CD ≥ II)	ND	No statistical differences in TO between laparoscopy and open group
van der Kaaij Br J Surg [4]	Unicentric Institutional Database Esophageal and gastric cancer	105	**Type of treatment:** total gastrectomy, subtotal gastrectomy, no resection **Neoadjuvant therapy:** none, chemotherapy, chemoradiotherapy	45.7%		Severe complications (CD ≥ II)	ND	Better overall survival in TO group 54 months vs 33 *p* = 0.018 3 years
Van der Werf Ann Surg [9]	Dutch Multicentric Database Esophageal or gastric cancer	2943		35%	Relationship between number of gastrectomies per year		More TO in high volume	TO is associated with survival 64% vs 45% (*p* < 0.002) 3 years
Carbonell-Morote Cir Esp [3]	Spanish unicentric database Gastric cancer	91	**Type of gastrectomy**: subtotal, total **Neoadjuvant treatment:** yes/no	34.1%	Relationship between TO and survival	≤ 15 lymph nodes examined	ND	Achievement of TO related to greater survival (TO/noTO) 50.5 months vs. 31.5 (*p* < 0.008) 5 years
Dal Cero Eur J Surg Oncol [10]	Spanish Multi-institutional Database (EURECCA) Gastric or gastro-oesophageal junction (GEJ) cancer	1293	**Type of gastrectomy:** subtotal, total **Neoadjuvant therapy:** chemotherapy, chemoradiotherapy **Surgical approach:** open, laparoscopy **Timing of surgery**: elective, emergency **Multivisceral resection:** no, yes	41.1%	< 65 years BMI 24–29.9 Weight loss < 5% ASA score I–II CCI = 0 Preoperative Hb ≥ 10 gr/dL Antrum-pylorus location Laurén´s mixed type Neoadjuvant chemotherapy Surgery performed in 2017 Laparoscopic surgery Elective surgery Surgery without multivisceral resection	Severe complications (CD ≥ II)	TO in community hospitals (19.9%) lower than in reference and high technology centers (44.0 and 36.1%) (*p* = 0.01)	Archievement of TO related to greater survival 72.79 (95%CI: 68.90–76.90) vs 53.48% (95%CI:49.93–57.28) 36 months *p* < 0.001
Sedłak Eur J Surg Oncol [11]	Polish Unicentric Database Gastric cancer	194	**Type of gastrectomy:** total gastrectomy, proximal gastrectomy, subtotal gastrectomy, esophagectomy with proximal gastrectomy **Perioperative chemotherapy:** yes, no	40.2%			ND	TO group 51% lower risk of death compared to patients without TO
Bolger Eur J Surg Oncol [12]	Two center Ireland Esophageal and gastric cancer	258	**Type of gastrectomy**: subtotal gastrectomy, total gastrectomy	37%	Age Neoadjuvant therapy Laparoscopy		ND	Minimally invasive surgery is associated with improved TO
Roh Sci Rep Nature [13]	Korean unicentric database	395	Type: of gastrectomy: robotic total gastrectomy (RTG) vs. laparoscopic total gastrectomy (LTG)	RTG: 70.3% LTG 75.7%		Severe complications	ND	
Spolverato J Surg Oncol [7]	International multi-institutional database Gastric adenocarcinoma	910		35.3%	Age *H. pylori* infection Family history of GC Diffuse subtype gastric adenocarcinoma Other than T1 Stage	≤ 15 lymph nodes	ND	Included chemotherapy inside TO
Cibulas Ann Surg Oncol [6]	National Cancer database Curative Gastrectomy	34,688	**Type of gastrectomy:** partial, subtotal, and total gastrectomy **Neoadjuvant therapy:** Chemotherapy receipt of neoadjuvant and/or adjuvant Chemotherapy for pT3-T4 and/or pN1-N2 disease **Surgical approach:** ND **Timing of surgery**: ND Mortality 30 days and mortality 90 days registered but not included on TO	23.8%	Race white better Insurance status private insurance Mean “Crow fly” distance Facility cancer program type better academic research Case-volume better very high Extent gastrectomy better partial Low T/N-stage No lymphovascular invasion No 30 and 90 days mortality	≤ 15 lymph nodes	Yes case-volume per year quartiles TO Low: 9.5% Intermediate: 18% High: 27.6% Very high: 44.9%	Achievement of TO related to greater survival (TO/noTO) 57% vs 38% TO attainment was significantly associated with reduced risk of death HR 0.82 *p* < 0.001
Levy Ann Surg [14]	Population-based clinical-pathological database	1836	Gastric	22%	Age Neoadjuvant surgery Low T-stage No GE junction tumor	< 15 lymph nodes examined Severe complications	ND	TO associated with survival 75% Vs 45% *p* < 0.001

*GC* gastric cancer, *TO* textbook outcome, *GE* gastroesophageal, *RTG* robotic total gastrectomy, *LTG* laparoscopic total gastrectomy

**Table 2:** Parameters included inTO

Author/year journal	Postoperative complication Clavien-Dindo	Length of stay	Mortality	Readmission	Lymph nodes examined > 15	Negative margins (R0)	Complete resection	Intraoperative complication	No reintervention	No readmission in ICU
Busweiler Br J Surg [1]	Yes CD ≥ II	Yes ≤ 21 days	Yes ≤ 30 days	Yes ≤ 30 days	Yes ≥ 15	Yes	Yes	Yes	Yes	Yes
Priego J Laparoend Adv Surg Tech [8]	Yes CD ≥ II	Yes ≤ 21 days	Yes ≤ 30 days	Yes	Yes > 15	Yes	Yes	No	Yes	Yes
van der Kaaij Br J Surg [4]	Yes CD ≥ II	Yes ≤ 21 days	Yes ≤ 30 days	Yes ≤ 30 days	Yes ≥ 15	Yes	Yes	Yes	Yes ≤ 30 days	Yes ≤ 30 days
Van der Werf Ann Surg [9]	Yes	Yes > 21 days	Yes	Yes	Yes ≥ 15	Yes	Yes	Yes	Yes	Yes
Carbonell-Morote Cir Esp [3]	Yes CD > IIIa	Yes < 21 days	Yes ≤ 30 days	Yes ≤ 30 days	Yes > 15	Yes	Yes	No	No	No
Dal Cero Eur J Surg Oncol [10]	Yes CD ≥ II	Yes ≤ 14 days	Yes ≤ 90 days	Yes ≤ 30 days	Yes ≥ 15	Yes	Yes	No	Yes ≤ 30 days	No
Sedłak Eur J Surg Oncol [11]	Yes DC ≥ II CCI ≥ 30	Yes ≤ 21 days	Yes ≤ 30 days	Yes	Yes ≥ 15	Yes	Yes	Yes	Yes ≤ 30 days	Yes
Bolger Eur J Surg Oncol [12]	Yes CD > II	Yes < 21 days	Yes ≤ 90 days	Yes ≤ 90 days	Yes ≥ 15	Yes	Yes	Yes	Yes	Yes
Levy Ann Surg [14]	NO severe complications	Yes	Yes	Yes	Yes ≥ 15	Yes	No	Yes	Yes	Yes
Roh Sci Rep Nature [13]	Yes CD ≥ II	Yes ≤ 21 days	Yes 30 days	Yes	Yes ≥ 15	Yes	No	Yes	Yes	Yes
Spolverato J Surg Oncol [7]	No	Yes	Yes	No	Yes ≥ 16	Yes	No	No	No	No
Cibulas Ann Surg Oncol [6]^a^	No	Yes < 12 days	No	Yes ≤ 30 days	Yes ≥ 15	Yes	No	No	No	No

^a^Included as TO parameter receipt of neoadjuvant and/or adjuvant chemotheraphy for pT3-T4 and/or pN1-N2 disease

The articles were included or rejected based on the pre-defined criteria and on the information obtained from the title and summary matched by three authors (SC, JMR, CV). Searches for duplicate series were performed, and in case of doubt, the article was read in full. The references of the selected articles were also checked, though no additional articles not included in the initial search were found.

The quality of the studies was assessed using the Newcastle–Ottawa scale (Table 3) [5]. Scores of 0–2 were considered poor quality, 3–5 fair, and 6–9 good or high. None of the studies were RCTs.

**Table 3:** Ottawa-Newcastle scale of the included studies

Study		Selection				Comparability	Outcomes			Total 9/9
	Type of study Prospective/retrospective	Representative of the exposed cohort	Selection of non-exposed cohort	Ascertainment of exposure	Outcome of interest not present at the start of the study	Comparability of cohorts on the basis of the design or analysis controlled for confounders	Assessment of outcomes	Sufficient follow-up time	Adequacy of follow-up and cohorts	
Busweiler [1]	Retrospective	*	*	*	*	*/-	-	*	*	7/9
Priego [8]	Retrospective	*	*	*	*	*/-	-	*	*	7/9
Van der Kaaij [4]	Retrospective	*	*	*	*	*/-	-	*	*	7/9
Van der Werf [9]	Retrospective	*	*	*	*	*/-	-	*	*	7/9
Carbonell-Morote [3]	Retrospective	*	*	*	*	*/-	-	*	*	7/9
Dal Cero [10]	Retrospective	*	*	*	*	*/-	-	*	*	7/9
Sedłak [11]	Retrospective	*	*	*	*	*/-	-	*	*	7/9
Bolger [12]	Retrospective	*	*	*	*	*/-	-	*	*	7/9
Levy [14]	Retrospective	*	*	*	*	*/-	-	*	*	7/9
Roh [13]	Retrospective	*	*	*	*	*/-	-	*	*	7/9
Spolverato [7]	Retrospective	*	*	*	*	*/-	-	-	-	5/9
Cibulas [6]	Retrospective	*	*	*	*	*/-	-	-	-	5/9

This systematic review has been registered in the Research Registry (reviewregistry1695).

## Results

**Fig. 1:**
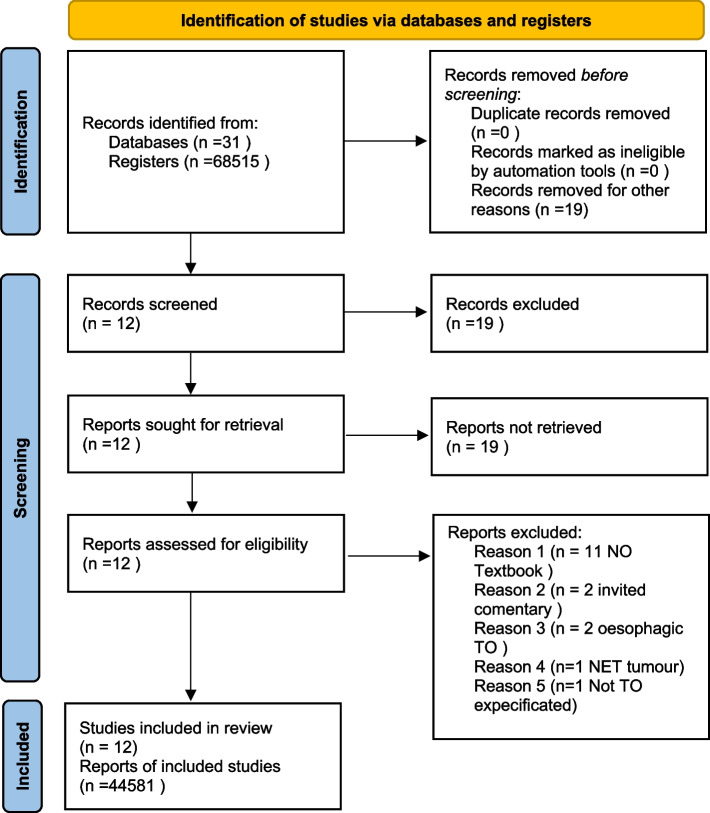
Algorithm PRISMA guidelines

The search yielded 31 articles. Nineteen of the articles were excluded, for the following reasons: 11 were not on TO, two were invited comments on articles about TO, two evaluated esophageal TO exclusively, one included only neuroendocrine gastric tumors, one evaluated TO after bariatric surgery, and another unspecified oncological TO. Figure 1 shows the PRISMA diagram (Fig. 1).

Ten studies were considered to be good quality according to the Ottawa-Newcastle scale and two were fair (Table 3). Due to the heterogeneity in the design of the studies and the variables used, we were unable to carry out a meta-analysis of the data.

Finally, the study focused on 12 articles including 44,581 patients who had undergone resection for gastric cancer and in whom TO had been measured. Six of the studies were multicenter, and these provided the largest number of cases (*N* = 41,606, 93.3% of the total), five were single-center (*N* = 881, 8.9%), and one was conducted at two centers (*N* = 258, 2.6%). In four studies, the results came from population databases (*N* = 39,589, 88.8%) [6].

The median number of patients who attained TOGS was 38.6% IQR (34.3–49.7). The rates of TOGS achieved ranged widely (22.7–75.7%) (1,17), but were between 34 and 45% in eight of the studies. Only two studies (16.7%) surpassed the threshold of 50% TO (18,19)—one single-center study in Korea and another in Spain. The median TOGS obtained in population studies (92.5% of all patients) was 27.9% (11,13,16,20) (Table 1).

### Parameters included in the assessment of TO

All the publications except one included mortality as a TO variable (16). The exception also provided the largest number of cases, although it reported mortality in the series at 30 days (1.4% TOGS group versus 4.7% non-TOGS group) and 90 days (2.3% TOGS versus 9.4% non-TOGS) and presented statistically significant differences in favor of the TOGS group [6]. Of the rest of the studies, three (25%) did not specify whether mortality was assessed at 30 or 90 days (1,20,21), six (50%) measured it at 30 postoperative days, and two (16.6%) at 90 days (3,9,12). The readmission rate was measured in ten articles (83.3%), although there was no consensus regarding the number of days required for its measurement: five (41.7%) assessed it at 30 days, one at 90 days, and in five (41.7%), the time point was not specified (2,3,10–12,14,17,18,20,21,23,24). Readmission was included as a TOGS variable in all but one study [7].

All the studies included the number of lymph nodes examined in the surgical specimen, and all but one (91.7%) applied a cutoff point ≥ 15; the exception established the cutoff point at > 16 nodes [7]. Complete resection was included in 66.7% of the studies, and R0 resection was included in all the articles reviewed (2,3,10–12,14,17,18,20,21,23,24).

Eight studies (66.7%) included the variables postoperative complications, readmission to the intensive care unit (ICU), and hospital stay in the definition of TOGS. Specifically, non-reoperation was included in nine (75%)—in three of these studies, this parameter was assessed at 30 days, and in six, the time point was not specified. Complications were measured according to the Clavien-Dindo (CD) scale in eight articles (66.7%) (3,10–12,14,17,23,24) using a CD score of ≥ II as a cutoff point to rule out TOGS. Two articles (16.6%) mentioned and included postoperative complications, but did not specify the classification used (1,20). In addition to the CD classification, one article used a Comprehensive Complication Index (CCI) score of ≥ 30 as a measure of complications [11].

Non-re-admission to the ICU was included for the evaluation of TOGS in eight (66.6%) of the studies. Length stay was included in ten studies, eight of which used the 75th percentile of the stay (≤ 21 days), one a stay of ≤ 14 days [3], and the other a stay of < 12 days [6]. Finally, the intraoperative complication variable was included in seven studies (58.3%). The rest of the variables that make up the TO are described in Table 2.

### Variables associated with obtaining TOGS

The parameters most frequently associated with the failure to achieve TOGS were fewer than 15 lymph nodes examined in the surgical specimen (five studies, 41.7%) and severe complications ≥ CD II (four studies, 33.3%). In three studies (25%), the variables that most influenced the non-achievement of TO were not specified.

In seven articles (58.3%), a significant increase in long-term survival was found in patients who obtained TO. Table 1 shows the rest of the parameters included in the review. Advanced age had a negative impact on obtaining TO (2,22). ASA < 3, Charlson < 2, and CCI = 0 were other factors significantly associated with obtaining TO (3,22).

In four articles, neoadjuvant treatment was positively associated with obtaining TO (1,2,8,16). Two were European, and the other two were from the USA. One of the US articles included neoadjuvant therapy as a TOGS parameter using data from a national database [6].

Other factors positively associated with obtaining TOGS were BMI 24–29.9, weight loss < 5% pre-surgery, preoperative hemoglobin ≥ 10 g/dL, location of the tumor in the antrum, laparoscopic surgery, and non-performance of multivisceral resection.

Finally, other factors associated with lower rates of achieving TOGS were tumors located in the esophagogastric junction and diffuse-type histology. No study found statistically significant differences with regard to gender (1,3,10,11,14,17,20,23).

## Discussion

TO is a tool that comprises a range of variables to assess the quality of care, and its use is currently increasing (1). This systematic review of TOGS yielded a median rate of 38.6%. Ten of the studies (83.3%) analyzed did not achieve a TOGS rate of 50%. Among the studies that surpassed this rate, the results of the only Asian series (a single-center Korean study) stand out, with a TOGS rate of 75.7% in 395 patients (18). The next highest rate was achieved in another single-center study in Spain, with a rate of 51.04% in 96 patients [8]. Multicenter studies and those that obtained data from national databases obtained even lower figures for TOGS (2,9,16). These data reopen the discussion on whether the results obtained in Asia and Western countries are comparable and highlight the drawbacks of using large population databases in which much data may be lost. The fact that other surgeries of similar complexity, such as pancreatic, liver, or colorectal surgery, obtain values close to 60% suggests that TO may be more difficult to achieve in the case of gastric cancer surgery (24,25). Probably, the inclusion of a higher number of parameters is one of the reasons for the lower rate of TO in this setting.

Although there is no internationally accepted definition of TOGS, the first article on TO in gastric surgery used ten parameters [1], a considerably higher number than the six initially described by Kolfschoten et al. for TO in colon cancer [15]. The ten variables originally described by Busweiler et al. for the definition of TOGS were complete resection, pathological R0, and > 15 lymph nodes in the surgical specimen, no intraoperative complications, no reintervention, no ICU readmission, no prolonged hospital stay (defined as the 75th percentile of stay, 21 days if applicable), no mortality or readmission at 30 days, and no severe complications, defined as CD ≥ II [1].

None of the studies published since Busweiler et al.’s initial report have included these ten parameters: the only variables included by all the studies in the review are mortality, number of nodes > 15, hospital stay, and obtaining R0. The concordance between the rest of the parameters was 91.7% for non-readmission at 30 days, 75% for CD ≥ II complications and non-reoperation, and 66.7% for complete resection and non-readmission to the ICU. Only one article included neoadjuvant therapy as an additional parameter [6].

Examination of 15 or more lymph nodes in the surgical specimen and severe complications CD ≥ II had the greatest specific weight for reducing the rate of TOGS (41.7% and 33.3%, respectively). The influence of these two variables on the TOGS rate, together with the fact that they were included in 100% and 75% of the series, respectively, support their inclusion in a consensus TOGS. Obtaining ≥ 15 lymph nodes is associated with a good surgical technique and a high level of engagement on the part of the pathologists. The type of lymphadenectomy (D2), the preparation of the specimen by the surgeon after resection and the use of indocyanine green are factors that can help to obtain this high number of nodes and thus improve TO levels [16]. As regards the inclusion of CD grade ≥ II complications, perhaps the use of only severe complications > CD IIIa would receive more widespread support, as complications of this grade are considered by many authors to be major [17]. The inclusion of parameters such as reoperation (a CD grade IIIb complication) and ICU readmission might be unnecessary if the CD classification is used: the reason for readmission to the ICU is almost always reoperation (CD IIIb) or failure of one or more organs (CD IVa-b), which are major complications in the CD classification and automatically rule out TOGS. Lastly, the R0 variable already includes the concept of complete resection, so we suggest that the latter variable may be superfluous in the TOGS assessment.

Hospital length stay was analyzed in 91.7% of the series included in our study and is another of the parameters that most influenced the achievement of TO [1]. In 72.7% of the studies, hospital stay was considered prolonged when it was greater than the 75th percentile, ranging from 12 to 21 days. However, the result of this quality indicator is highly dependent on both the implementation of ERAS protocols and the appearance of major complications and so the inclusion of this parameter in the TOGS has been questioned (29–31). The 30-day readmission rate was included in 91.7% of the studies reviewed, although there is a direct relationship between early discharge and readmission [18]; therefore, the measurement of these variables at 90 days, as reported by some authors, would improve the measurement of TOGS, since it is considered that around a third of patients are readmitted at a point later than 30 days post-surgery [19].

Gastrectomy for cancer is a major surgery with a significant morbidity rate that ranges from 4 to 45% according to series (32,34), although there is no accepted standard definition of severe postoperative complications. A growing number of studies show that the decrease in morbidity and mortality seems to be associated with the volume of gastrectomies performed annually (35–39).

Postoperative mortality after surgery for gastric cancer continues to be high, but it varies significantly between series (2–10%) (2,32,40). Although postoperative mortality was analyzed in all the series, it was only measured at 90 days in three studies (2,16,22). It is important that mortality be evaluated 90 days postoperatively, since there are notable differences between the rates at 30 or 90 days (32,41,42).

The series included in our study refer to different parameters that influenced the achievement of TOGS, such as age, ASA classification and Charlson index, neoadjuvant therapy, tumor location, histological type, BMI, preoperative hemoglobin, type of approach, and association with multivisceral resections. Some of these variables require further analysis and could be independent factors associated with the achievement of TOGS.

Although the association between advanced age and postoperative morbidity and mortality after gastric surgery is not well defined, it has been reported in a growing number of studies (43–47). In 36.3% of the studies included here, a direct correlation was found between age and the possibility of obtaining TO, although no definite cutoff point was established in the series included (2,18,24). A report by the Dutch Upper Gastrointestinal Cancer Audit (DUCA) nationwide registry showed a trend towards significance for an association between age 70 and older and postoperative 30-day or in-hospital mortality (OR 1.56; 95% CI 0.99 to 2.46) (22). Some studies have reported a statistically significant increase in postoperative mortality from age 75 onwards [20]. A Japanese study of 327,642 patients undergoing major abdominal surgery (including gastric surgery) concluded that mortality increased with age in all procedures and that respiratory complications such as pneumonia were a key factor in mortality in this subgroup of elderly patients (> 80 years) (40,46,47). In the light of the above, we consider that TOGS should be adjusted according to the age of the patient. The ASA classification and the Charlson index are risk factors for morbidity and mortality that are usually correlated with age. ASA grade < 3 and Charlson index < 2 were significantly associated with obtaining TOGS in two articles. However, the validity of these parameters has been questioned because they may be affected by interobserver variability (50,51).

The administration of neoadjuvant chemotherapy, and specifically the perioperative FLOT scheme (fluoracil, oxaliplatin, leucovorin, docetaxel), has shown its beneficial role in terms of survival in cases of locally advanced gastric cancer and with positive lymph nodes, but it is routinely administered only in European countries [12]. Therefore, it is rarely included in studies of TOGS (2,18,24). On the other hand, the administration of neoadjuvant chemotherapy was associated with greater morbidity and mortality, although this assessment has been carried out with the MAGIC scheme, which is less toxic (46,52). One might speculate that patients receiving neoadjuvant therapy are a selected group of patients who have advanced tumors but are less frail and have a good status performance (40,46). For all these reasons, neoadjuvant treatment should be included in the assessment of TOGS.

As regards the surgical approach, the meta-analyses carried out do not report differences in survival, morbidity, and oncological results between laparoscopic and open gastrectomy, although the laparoscopic approach presents advantages in terms of earlier diet food intake, less surgical site infection, and shorter hospital stay [21]. Perhaps this is why in our study the series with the highest proportion of laparoscopic surgeries are the ones that obtain the highest TO rates (2,18,21).

In our review, the location of the tumor in the esophagogastric junction and diffuse histology were associated with a lower probability of obtaining TO. Perhaps the fact that this tumor location requires a total gastrectomy increases morbidity and mortality, although this supposition is not borne out by the literature; several studies comparing total and subtotal gastrectomy have found no differences in terms of mortality, blood loss, or hospital stay (53–55). In contrast, there seems to be a correlation between the number of lymph nodes in the surgical specimen and intra-abdominal collections in the postoperative period, which is higher in total gastrectomy (53,56). Although diffuse histology is associated with a worse prognosis, it tends to occur in younger patients who usually have a higher probability of TO [22]. The weight of these two variables should be studied in greater detail in future studies of TO.

Other variables positively associated with achieving TO in some of the series studied were BMI 24–29.9, weight loss < 5% pre-surgery, preoperative hemoglobin ≥ 10 gr/dL, and no multivisceral resection [12, 13]. Gender was not associated with the achievement of TO in any of the studies reviewed (1–3,10–12, 14,17,18,20,23,24).

Achieving TO was independently associated with a statistically significant increase in survival. Six of our studies (54.5%) found a survival benefit when TO was reached, with a median survival in the TO group almost 20 months higher than in the non-TO group.

The main limitation of our study was the heterogeneity of parameters used for the evaluation of TOGS in the articles included. This meant that comparison between them is almost impossible, and we were unable to perform a meta-analysis.

In conclusion, we believe that TOGS needs to be standardized in order to be able to carry out comparisons between groups. We propose the following six parameters for creating a consensus definition of TOGS: > 15 lymph nodes in the surgical specimen, R0 resection, absence of major complications (CD > IIIa) measured at 90 days, length of stay (75th percentile), 90-day mortality, and 90-day readmission. However, the analysis of other parameters such as age or the diversity of preoperative treatment, which in some countries includes neoadjuvant therapy, suggests that this basic definition of TOGS should be adjusted to incorporate these variables.

## Data Availability

Non-applicable, since this is a bibliographical review.

## Abbreviations

TO: Textbook outcome
GS: Gastric surgery
TOGS: Textbook outcome gastric surgery
CD: Clavien-Dindo
ICU: Intensive care unit
ASA: American Society of Anesthesiologist
CCI: Comprehensive Complication Index
US: United States
DUCA: Dutch Upper Gastrointestinal Cancer Audit
